# Resveratrol and Its Analogs: Potent Agents to Reverse Epithelial-to-Mesenchymal Transition in Tumors

**DOI:** 10.3389/fonc.2021.644134

**Published:** 2021-04-16

**Authors:** Kaibo Guo, Yuqian Feng, Xueer Zheng, Leitao Sun, Harpreet S. Wasan, Shanming Ruan, Minhe Shen

**Affiliations:** ^1^ The First Clinical Medical College of Zhejiang Chinese Medical University, Hangzhou, China; ^2^ Department of Medical Oncology, The First Affiliated Hospital of Zhejiang Chinese Medical University, Hangzhou, China; ^3^ Department of Cancer Medicine, Hammersmith Hospital, Imperial College Healthcare NHS Trust, London, United Kingdom

**Keywords:** epithelial-to-mesenchymal transition, metastasis, chemoresistance, cancer cell stemness, resveratrol

## Abstract

Epithelial-to-mesenchymal transition (EMT), a complicated program through which polarized epithelial cells acquire motile mesothelial traits, is regulated by tumor microenvironment. EMT is involved in tumor progression, invasion and metastasis *via* reconstructing the cytoskeleton and degrading the tumor basement membrane. Accumulating evidence shows that resveratrol, as a non-flavonoid polyphenol, can reverse EMT and inhibit invasion and migration of human tumors *via* diverse mechanisms and signaling pathways. In the present review, we will summarize the detailed mechanisms and pathways by which resveratrol and its analogs (e.g. Triacetyl resveratrol, 3,5,4’-Trimethoxystilbene) might regulate the EMT process in cancer cells to better understand their potential as novel anti-tumor agents. Resveratrol can also reverse chemoresistance *via* EMT inhibition and improvement of the antiproliferative effects of conventional treatments. Therefore, resveratrol and its analogs have the potential to become novel adjunctive agents to inhibit cancer metastasis, which might be partly related to their blocking of the EMT process.

## Introduction

In the past 10 years, a large number of studies have been conducted to determine cellular and molecular mechanisms in cancer invasion and metastasis (1). Epithelial-to-mesenchymal transition (EMT) has been widely reported to promote the acquisition of metastatic properties for tumor cells by enhancing mobility and invasion (2). EMT, as an invasive phenotype, is a reversible cellular program in which cells shed their epithelial features and adopt mesothelial traits (3), generating both morphological and molecular changes (4). Moreover, EMT is not only associated with cancer cell proliferation, invasion and metastasis, but also with chemoresistance and cancer cell stemness. EMT is known as the initial step of the development of metastasis (5), existing in various tumors such as lung, pancreatic, liver and prostate carcinoma (6–9). Resveratrol (RES) and its analogs reverse EMT to inhibit metastasis and mitigate chemoresistance *via* different pathways and mechanisms in human tumors (**Tables 1** and **2**). In the present review, we focus on summarizing and analyzing the mechanisms by which RES and its analogs affect EMT in different cancers, supporting their potential to be promising therapeutic agents.

**Table 1:** Summary of mechanisms by which resveratrol inhibits the EMT process.

Disease condition	Main mechanism	Ref.year
Gastric cancer	-reversing doxorubicin resistance by inhibiting mesenchymal markers (β-catenin and vimentin) through modulating PTEN/AKT signaling pathway	(10) 2017
	-prevention of EMT via down-regulating MALAT1	(11) 2019
	-suppression of hedgehog signaling pathway	(12) 2015
	-declining HIF-1α protein levels caused by hypoxia	(13) 2020
	-targeting gastric-cancer-derived mesenchymal stem cells -inactivating the Wnt/β-catenin signaling	(14) 2020
Colorectal cancer	-down-regulating the expression of Slug and vimentin -overexpression of E-cadherin and claudin-2 -blocking 5-FU-induced EMT by down-regulation of NF-κB activation -down-regulation of MMP-2 and MMP-9	(15) 2015
	-suppressing EMT through TGF-β1/Smads signaling pathway mediated Snail/E-cadherin expression	(16) 2015
	-down-regulation of MMP-9 and CXCR4 -down-expression of CSC markers (CD133, CD44 and ALDH1) -inhibiting the TNF-β-induced EMT by prevention of the FAK/NF-κB activation	(17, 18) 2018, 2019
	-prevention of EMT via AKT/GSK3β/Snail signaling pathway	(19) 2019
	-inhibiting EMT by increasing miR-200c expression	(20) 2017
Pancreatic cancer	-decrement of markers of EMT (ZEB-1, Slug and Snail) in CSCs	(21) 2011
	-preventing the expression of uPA and MMP2 -down-expression of HIF-α -inhibited hypoxia-mediated activation of the Hedgehog signaling pathway	(22) 2016
	-suppression of the PI3K/AKT/NF-κB signaling	(23) 2013
	-blocking hypoxia-induced pancreatic stellate cells (PSCs) activation -inhibiting the interaction between PSCs and pancreatic cancer cells	(24) 2020
	-blocking EMT process via the inhibition of NAF-1	(25) 2020
Cholangiocarcinoma	-decreasing the secretion (IL-6) of CAFs -reverting N-to E-cadherin switch by induction of autophagy (LC3-II/LC3I) in the incubation with CAFs-CM	(26) 2018
Breast cancer	-down-expression of mesenchymal markers (Fibronectin 1 and Vimentin) -decreasing the expression of ANGPTL4 and CXCL8 mRNA levels -antagonizing TGF-β signaling by activating SIRT7 deacetylase activity toward SMAD4 degradation	(27) 2017
	-reversing TGF-β1-induced EMT through the PI3K/AKT, Smad, and MMP Pathways	(28) 2019
	-preventing EGF-induced EMT by inhibiting Na+ channel expression	(29) 2019
	-inhibition of EGF-induced EMT by prevention of ERK activation	(30) 2011
	-inhibiting YB-1 phosphorylation induced by LPA and blocking EZH2/amphiregulin signaling axis	(31) 2019
	-inhibiting EMT via induction of Rad9	(32) 2019
	-promoting the epithelial-type alternative splicing of Cd44, Enah, and FGFR2 pre-mRNAs by upregulating KHSRP and hnRNPA1 expression	(33) 2017
	-promoting sensitization to doxorubicin by inhibiting EMT through modulating SIRT1/β-catenin signaling	(34) 2019
	-overcoming acquired tamoxifen resistance by reversing EMT through suppressing endogenous TGF-β1 production and Smad phosphorylation	(35) 2013
	-reduction of MK-2206(AKT inhibitor)-induced EMT via inducing b-TrCP-mediated Twist1 degradation	(36) 2016
	-enhancing the sensitivity of FL118 in triple-negative breast cancer cell lines through suppression of EMT process	(37) 2021
Lung cancer	-suppression of TGF-β1-induced EMT via decreasing ROS and inhibiting mitochondrial functions	(38, 39) 2018, 2013
	-inhibition of EMT by prevention of miR-520h-mediated PP2A/C-AKT-FOXC2 signaling pathway	(40) 2013
	-reversing hypoxia-induced EMT by abrogating the effect of PIASy and regulating SIRT1 Transcription	(41) 2013
Ovarian cancer	-inhibiting Cisplatin-mediated EMT by reducing ERK activation	(42) 2014
	-suppressing norepinephrine-induced EMT through the interference of a Src and HIF-1α/hTERT/Slug signaling cascade	(43) 2016
Cervical cancer	-preventing the EMT process by inhibiting STAT3^Tyr705^ phosphorylation	(44) 2020
Prostate cancer	-inhibition of EMT via blocking TRPM7 channel activity	(45) 2018
	-interfering the TRAF6/NF-κB/Slug axis	(46) 2020
	-suppression of LPS-induced EMT through inhibiting the Hedgehog signaling pathway	(47) 2014
	-preventing DHT-induced EMT through interfering with the AR and CXCR4 pathway	(48) 2019
	-blocking HGF-mediated interplay between the stroma and epithelium	(49) 2020
Bladder cancer	-attenuating CSE-induced EMT via suppression of STAT3/Twist1	(50) 2019
Glioblastoma multiforme	-down-expression of Bmi1 and Sox2 -decreasing TGF-β1-induced Smad/α-SMA pathway	(51) 2019
	-disturbing Wnt/β-catenin pathway in GSCs	(52) 2017
Pituitary adenoma	-down-regulation of the expression of CCNB1	(53) 2019
Head and neck cancer	-down-expression of Oct4, Nanog, and Nestin -down-regulating the expression of Slug, ZEB1, N-cadherin and vimentin	(54) 2012
Oral squamous cell carcinoma	-prevention the expression of Smad2/3 -down-regulating the expression of EMT markers (Slug, Snail and N-cadherin) -induction the expression of E-cadherin	(55) 2018
	-reversing the up-regulation of RCP-induced ZEB1 and MT1-MMP expression	(56) 2020
Nasopharyngeal Carcinoma	-impeding EMT through p53 activation in CSCs	(57) 2013
Melanoma	-inhibition of LPS-induced EMT through the down-regulation of NF-κB activity	(58) 2012
	-preventing MRC5 fibroblast SASP-related protumoral effects on melanoma cells	(59) 2017
Osteosarcoma	-promoting HIF-1α protein degradation	(60) 2015

AKT, protein kinase B; ALDH, aldehyde dehydrogenase; ANGPTL4, angiopoietin-like protein 4; AR, androgen receptor; CAFs, cancer associated fibroblasts; CAFs-CM, conditioned medium from CAFs; CCNB1, cyclin B1; CSCs, cancer stem cells; CSE, cigarette smoke extract; CXCL8, chemokine C-X-C motif ligand 8; CXCR4, chemokine C-X-C motif receptor 4; DHT, dihydrotestosterone; EGF, epidermal growth factor; EMT, epithelial-mesenchymal transition; EMT-TFs, EMT-inducing transcription factors; ERK, extracellular signal-regulated kinase; EZH2, enhancer of zeste homolog 2; FAK, focal adhesion kinase; FGFR, fibroblast growth factor receptor; FOXC2, forkhead box C2; 5-FU, 5-Fluorouracil; GCSs, glioma stem cells; GSK, glycogen synthase kinase; HIF-1, hypoxia-inducible factor-1; hTERT, human telomerase reverse transcriptase; HGF, hepatocyte growth factor; IL-6, interleukin-6; HKSRP, hnRNPK-homology splicing regulatory protein; LC3-II/LC3I, light chain3; LPA, lysophosphatidic acid; LPS, lipopolysaccharide; MALAT1, metastasis-associated lung adenocarcinoma transcript 1; miRNAs, miRs ,microRNAs; MMP, matrix metalloproteinase; NAF-1, Nutrient-deprivation autophagy factor-1; NF-κb, nuclear factor-κB; Oct4, octamer-binding transcription factor 4; PI3K, phosphatidylinositol 3-kinase; PIASy, protein inhibitor of activated STAT 4; PTEN, phosphatase and tensin homolog deleted on chromosome ten; PSCs, pancreatic stellate cells; ROS, reactive oxygen species; RCP, rab coupling protein; SASP, senescence-associated secretory phenotype; SIRT1 silent information regulator 1; SIRT7, sirtuin 7; STAT3, signal transducer and activator of transcription 3;TGF-β transforming growth factor-β; TNF-β, tumor necrosis factor-β TRAF6, TNF-receptor associated factor 6; TrCP, transducing repeats containing proteins; TRPM transient receptor potential melastatin; uPA, urokinase-type plasminogen; YB-1, Y-box binding protein 1; ZEB, zinc finger Ebox binding homeobox.

**Table 2:** Summary of mechanisms by which resveratrol descendant inhibits the EMT process.

Drug(Disease condition)	Main mechanism	Ref.year
Resveratrol analogues (Ovarian cancer)	-inhibiting AKT and MAPK signaling and reversing EMT induced by IL-6 and EGF	(61) 2017
3,5,4’-Trimethoxystilbene (Breast cancer) Triacetyl resveratrol (Pancreatic cancer)	-up-regulation of E-cadherin expression -elevating the phosphorylation and ubiquitination of β-catenin by employing the PI3K/AKT/GSK3β-dependent pathway -suppressing Zeb1 3’UTRluciferase activity through the upregulation of miR-200	(62) 2017 (63) 2013

AKT, protein kinase B; EMT, epithelial-mesenchymal transition; EGF, epidermal growth factor; GSK3β, glycogen synthase kinase 3β; MAPK, mitogen-activated protein kinase; PI3K, phosphatidylinositol 3-kinase.

## The EMT Process

EMT is an evolutionarily conserved process whereby epithelial cells lose cell junctions and apical-basal polarity, and are eventually converted into migratory mesenchymal phenotype (64). As a key program, EMT participates in the development of embryogenesis such as gastrulation and tissue morphogenesis, as well as wound healing of adult (65). In addition, EMT has been related to different cancer functions, especially for tumor invasion and metastasis, tumor stemness and resistance to therapy (66). Correspondingly, the resulting mesenchymal cells after migration also can reverse the process, known as mesenchymal-to-epithelial transition (MET), transforming epithelial cells to colonize a particular location (67).

**Figure 1:**
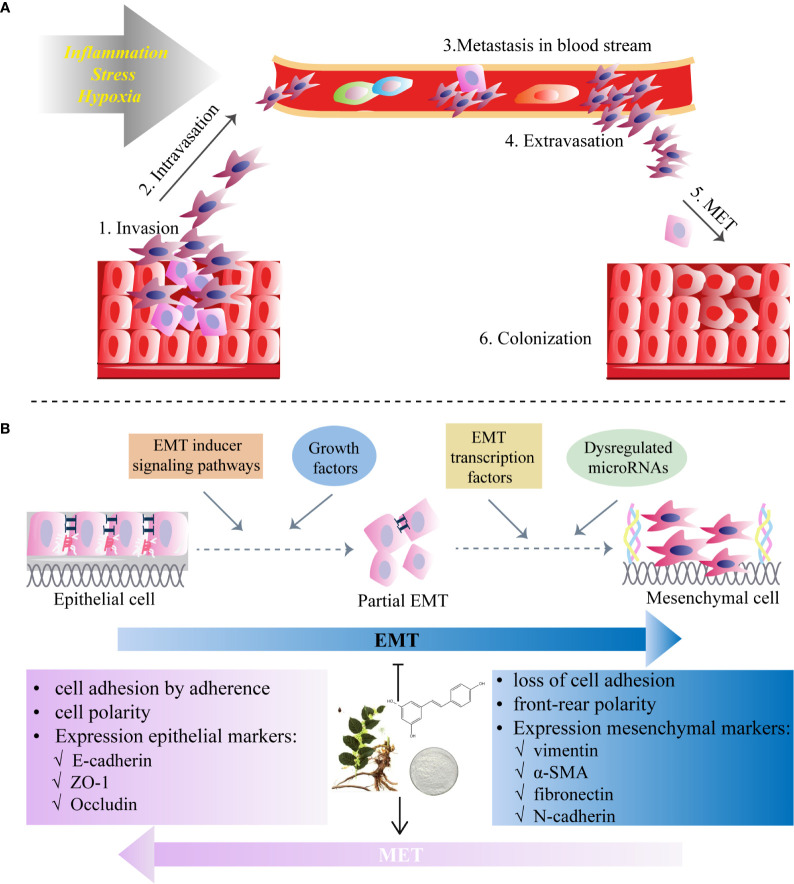
Epithelial-to-mesenchymal transition (EMT) programme in cancers and the inhibitory role of RES. **(A)** Invasion and migration initiated by the EMT process due to the factors (e.g., inflammation, stress and hypoxia) affect tumor microenvironment. **(B)** Resveratrol inhibits EMT which starts from diverse processes (EMT inducer signaling pathways, growth factors, dysregulation of microRNAs and EMT transcription factors) and by which polarized epithelial cells acquire motile mesothelial features. The EMT process is characterized by the loss of cell-cell contacts *via* downregulation of epithelial markers (i.e. E-cadherin in adherent junctions, zonula occludens-1 (ZO-1) in tight junctions and desmoplakin in desmosomes) and upregulation of mesenchymal markers (N-cadherin, vimentin, fibronectin and α-smooth muscle actin (α-SMA)). RES may also induce mesenchymal-to-epithelial transition (MET), a reversal of the EMT process.

In the EMT process, polarized and immobile epithelial cells with cobblestone-like appearance acquire mesothelial traits with a spindle-like shape and increased cellular motility, by which they are separated from the epithelial basement membrane and invade through surrounding tissues (68) (**Figures 1A, B**). Then tumor cells intravasate into blood vessels and circulate in the bloodstream, eventually leading to extravasation to distant organs and form metastatic lesions (69) (**Figure 1B**). This process starts from dissociating of cell-cell connection *via* the loss of epithelial phenotype including adhesion molecule (E-cadherin), gap- and tight junctions (claudin-2) and desmoplakin (70). With the overexpression of mesenchymal markers (71) such as N-cadherin (N-cad), vimentin (VIM), fibronectin (FN) and α-smooth muscle actin (α-SMA), tumor cells could reconstruct the cytoskeleton, degrade the basement membrane, and remodel extracellular matrix (ECM) by inducing matrix metalloproteinases (MMPs), such as MMP-2 and MMP-9 (72), underlying metastasis to distant organs, stemness maintenance and reversion of chemoresistance (73).

Several critical EMT-inducing transcription factors (EMT-TFs), which include zinc-finger E-box binding homeobox 1 (ZEB1), ZEB2 (also known as SIp1), Snail1 (also known as Snail), Slug (also known as Snail2) and Twist-related protein 1(Twist1; also known as Twist), have been demonstrated to play significant regulatory roles in the EMT program of cancer cells (74). In addition to activating the classic EMT traits and promoting tumor metastasis, EMT-TFs are associated with the induction to many other features, like maintenance of cancer stem cells (CSCs) and resistance to therapeutic drugs (75). CSCs are referred to tumor cells with the ability for self-renewal and recapitulation of the tumor heterogeneity (76). The EMT process, as a key regulator of the cancer cell stemness, provides a chance to research the nature of intratumoral heterogeneity and offers a potential mechanistic basis for the resistance of anti-cancer drugs (77).

Transforming growth factor-β (TGF-β1), a secret multifunctional cytokine, is participated in the EMT process, regulating EMT-TFs (78). TGF-β1 can contribute to the EMT program through both Smad-mediated and non-Smad signaling. Smad2 and Smad3 are phosphorylated by TGF-β1 signals, then form hetero trimeric complexes about Smad4, translocating into the nucleus, activating the expression level of EMT-TFs, and cooperating with these transcription factors to regulate EMT-related genes (79). In addition, non-Smad pathways are also involved in the TGF-β1-induced EMT process, such as phosphatidylinositol 3-kinase (PI3K)/AKT/mammalian TOR complex 1 (mTORC1), tumor necrosis factor receptor-associated factor 6 (TRAF6)/TGF β-Activated Kinase 1 (TAK1), and Wnt/β-catenin signaling (80–82).

## Resveratrol: A Bioactive Compound

Resveratrol (3,5,4’-trihydroxy-trans-stilbene), initially isolated in 1939 from Veratrum grandiflorum by Takaoka (83), is a phenolic substance present in various plants, including grapes, soy, peanuts and berries as well as the roots of Polygonum cuspidatum, a traditional medicine in China. It has been found to exert diverse pharmacological effects involving anti-inflammation, anti-oxidation, anti-aging, anti-diabetes, anti-obesity, anti-cancer, cardioprotection and neuroprotection (84–86). Interestingly, resveratrol (RES) can regulate various signaling pathways and target diverse effector molecules, as well as alter phenotypes of disease models. RES acts as a natural autophagy regulator for prevention and treatment of Alzheimer’s disease (87). RES also has shown promising effects on inflammatory bowel disease, as its anti-inflammatory and anti-oxidant activity (88). Moreover, RES moderately diminishes systolic blood pressure and blood glucose, exerts numerous vasculoprotective effects by suppressing vascular smooth muscle cell proliferation (89). Increasing evidence revealed that RES had the wide range of preventive and therapeutic roles against various types of tumor (90), including colorectal, breast and lung cancers (15, 23, 27), and involved various phases of cancer development, such as EMT. RES has been reported to regulate numerous functional proteins, including growth factors, inflammatory cytokines, transcription factors, as well as free radicals, which are all participated in the initiation and progression of human cancer (91).

**Figure 2:**
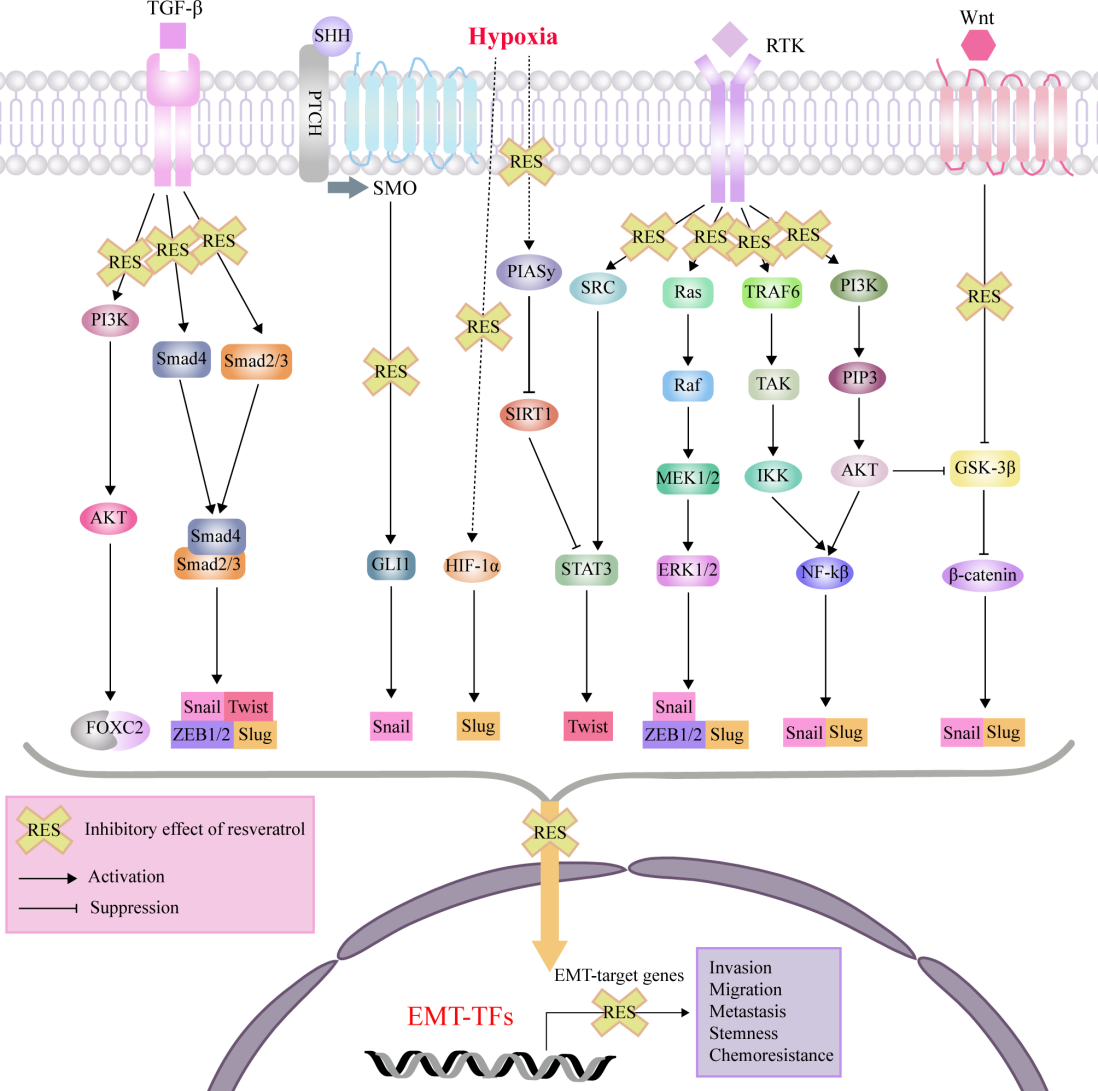
Mechanisms of effects of RES through interference with signaling pathways affecting EMT, invasion, migration, metastasis, tumor stemness and chemoresistance. protein kinase B (AKT); epithelial-mesenchymal transition (EMT); extracellular signal-regulated kinase (ERK); forkhead box C2 (FOXC2); glioma-associated oncogene 1 (GLI1); glycogen synthase kinase 3β (GSK-3β); hypoxia-inducible factor-1 (HIF-1); inhibitor of nuclear factor kappa-B kinase (IKK); nuclear factor-κB (NF-κB); MAP kinse-ERK kinase (MEK); protein inhibitor of activated STAT 4 (PIASy); patched (PTCH); phosphatidylinositol 3-kinase (PI3K); plasma membrane intrinsic protein 3 (PIP3); receptor tyrosine kinases (RTK); sonic hedgehog (SHH); sirtuin1(SIRT1); smoothened (SMO); signal transducer and activator of transcription 3 (STAT3); transforming growth factor-β (TGF-β); TGF beta-Activated Kinase (TAK); TNF-receptor associated factor 6 (TRAF6); zinc finger E-box binding homeobox (ZEB).

RES exerts its anti-cancer and chemosensitivity effects through regulation of the tumor microenvironment and the malignant biological behaviors, including levels of reactive oxygen species (ROS), proliferation, antiapoptosis, invasion, migration, EMT progress, and stemness (92). As an effective antioxidant, RES can scavenge intracellular ROS *via* suppression of extracellular signal-regulated kinase 1/2 (ERK1/2) and p38 mitogen-activated protein kinase (p38 MAPK) (93). For the proliferation and viability of tumor cells, RES can induce cell cycle arrest and apoptosis by suppressing Wnt/β-cantenin signaling and downregulating antiapoptotic proteins (Bcl-2 and Bcl-XL), respectively (94, 95). Recent studies have been reported that RES can directly target cytokines, and cellular signaling molecules such as, forkhead box C2 (FOXC2), forkhead box O1 (FOXO1), human telomerase reverse transcriptase (hTERT), glioma-associated oncogene 1 (GLI1), β-catenin, ERK1/2 and nuclear factor-κB (NF-κB), which promote the EMT process in different disorders with a variety of cellular signaling pathways (**Figure 2**).

## Effects of Resveratrol on EMT in Human Cancers

### Digestive Cancers

#### Gastric Cancer

There have been accumulating clinical trials which demonstrated that doxorubicin (DOX)-based regimens failed to attain the favorable survival goal and brought about some side effects for patients with gastric cancer (96, 97). EMT participates not only in the cancer metastasis but also in the appearance of chemoresistance (98). EMT-mediated drug resistance is modulated by several signalings, among which PI3K/AKT pathway is of high attention (99). RES can reverse DOX resistance *via* the inhibition of EMT by suppressing mesenchymal markers (β-catenin and vimentin) through regulating phosphatase and tensin homolog deleted on chromosome ten (PTEN)/AKT signaling pathway (10). Besides RES can further promote apoptosis (10). That’s say, RES combined with DOX can not only suppress the growth of gastric cancer cells, but also alleviate the resistance of DOX and prohibit cell migration through the reverse of EMT by regulating PTEN/AKT pathway.

Metastasis-associated lung adenocarcinoma transcript 1 (MALAT1), which is also named by nuclear-enriched abundant transcript 2, has a strong function in EMT, proliferation, apoptosis and so on (100). In Yang’s gastric cancer cell model, RES was found to prevent EMT by down-regulating MALAT1 (11), which provided new evidence for anticancer mechanism of RES *in vitro*.

It is believed that the hedgehog signaling pathway could exert a critical role in vertebrate growth. e.g. the homeostatic process and tumorigenesis (101). Gli-1 is considered as a marker when the hedgehog signaling pathway activates abnormally (102). RES can prevent hypoxia-induced gastric cancer invasion and EMT by suppressing the hedgehog signaling as proved that RES can reduce the expression of Gli-1, Snail and N-cadherin, while enhancing the expression of E-cadherin (12, 13). The mechanism may be explained that Gli-1 directly induced Snail which marks the process of EMT (103), so the expression of Snail is decreased as soon as Gli-1 is suppressed. Moreover, RES can target mesenchymal stem cells derived from gastric cancer by inactivating the Wnt/β-catenin signaling pathway (14).

#### Colorectal Cancer

5-FU is routinely applied to the treatment of colorectal cancer, but its toxicity and ineffectiveness in many patients limit its wide application. Furthermore, over 50% of patients show resistance to 5-FU in the therapeutic course (15). RES has been found to inactive the NF-κB signaling pathway potently in earlier researches (104–106). The NF-κB signaling pathway not only closely links to inflammation and cancer (107), but also propels the process of EMT and metastasis (17). RES significantly attenuated 5-FU-induced EMT by down-regulation of NF-κB activation, which is proved by the evidence that RES suppresses EMT factors (down-regulating the expression of Slug and vimentin as well as up-regulating of E-cadherin) and overexpresses the gap and tight junctions (claudin-2) in colorectal cancer cells (15). Based on these findings, RES may perform as an anticancer agent in colorectal cancer.

The process of EMT can be triggered by many cytokines such as TGF-β1, HGF and so on (108–110). Different signaling pathways and mechanisms connected with EMT were studied, like TGF-β1/Smads pathway Qing Ji’s team focused on (16). They found that RES exerted an inhibitory role on EMT of colorectal cancer cells both *in vivo* and *in vitro*. RES has the potential to suppress EMT *via* TGF-β1/Smads signaling, as proved by the up-regulating of E-cadherin and the down-regulating of Snail and vimentin (16). Snail, a TGF-β1/Smad signaling pathway modulated gene (111, 112), restrains the E-cadherin expression and triggers the EMT process (16). Furthermore, they also found that with the treatment of RES, TGF-β1-induced MMP2 and MMP9 decreased through expression analysis (16). These findings may explain how RES inhibit invasion and metastases.

Previous studies reported that inflammatory cytokines like members of the tumor necrosis factor (TNF)-superfamily could change the tumor microenvironment and promote the progression of colorectal cancer through activation of EMT. These pro-inflammatory mediators are modulated *via* the transcription factor NF-κB (113–116). It has also been reported that focal adhesion kinase (FAK) associated with NF-κB regulates the cancer cells capacity of survival, invasiveness, and metastasis (117–120). Buhrmann and co-workers showed that RES inhibited the TNF-β-induced EMT as demonstrated by suppressing EMT factors (up-modulating vimentin and slug, down-modulating E-cadherin) in colorectal cancer cells through preventing the FAK/NF-κB activation as proved by down-regulation of MMP-9 and C-X-C motif chemokine receptor 4 (CXCR4) which are the NF-κB-dependent of tumor-promoting factors (17, 18). Moreover, it has been reported that cancer stem cells (CSCs) had stem cell characteristics like pluripotency, self-renewal, invasion, motility (121–124). CD133, CD44 and aldehyde dehydrogenase gene 1 (ALDH1), as molecules associate with CSCs, are widely used to mark CSCs (125, 126). The researchers also found that RES restrained CSC-like phenotype (CD133, CD44, ALDH1) to suppress the formation of CSCs (18). Targeting both stemness of cells and EMT underscores the potential of RES to develop a new therapeutic strategy for colorectal cancer.

Serine/threonine kinase (AKT) is closely linked to EMT (80) and is also related to some biological and pathological proceedings, like angiogenesis, invasion, and metastasis (127). Moreover, it has been found that the glycogen synthase kinase (GSK)−3β pathway often involves in the process of EMT because it is the downstream pathway of AKT (128). Furthermore, the AKT/GSK−3β signaling pathway can modulate the expression of Snail and promote EMT in some cancer cells (129, 130). Li and colleagues revealed that RES prevented EMT *via* AKT/GSK3β/Snail signaling pathway in colon cancer, and they observed markedly increased E−cadherin, whilst the decreased expression of N−cadherin both *in vitro* and *in vivo (* 19). Concordantly, the activation of phospho (p)−AKT1, p−GSK−3β, and Snail also decreased (19). Taken together, these findings demonstrate that RES may provide new treatment for colon cancer.

More recently, it has been indicated that the miR-200 family exerts a vital role on the modulation of the EMT process (131). ZEB1, considered as the target gene of miR-200 family, can repress E-cadherin to promote EMT process, subsequently bring about cancer progression (132). Based on the data of Dermani’s team, RES could inhibit EMT by enhancing miR-200c expression in colorectal cancer cells, its mechanism might be explained as targeting ZEB1 and vimentin expression (20).

Up to now, there are involving some clinical study of resveratrol in cancer treatment. It was reported that long non-coding (Lnc) MALAT1 was upregulated in colorectal cancer tissues and RES inhibits invasion and metastasis *via* Lnc MALAT1 mediated Wnt/β-catenin signal pathway (133). Patel et al. found that daily oral doses of resveratrol at 0.5 or 1.0g makes the resected tissues keep resveratrol and its metabolite resveratrol-3-*O*-glucuronide with proper concentrations, exerting antitumor effects for patients with colorectal cancer (134). Moreover, Howells et al. conducted a phase I randomized, double-blind pilot study and assessed the safety, pharmacokinetics, and pharmacodynamics of micronized resveratrol (SRT501) for colorectal cancer patients with patients with hepatic metastases. They revealed that SRT501 was well tolerated and cleaved caspase-3, a marker of apoptosis, significantly increased by 39% in metastatic hepatic tissues from the SRT501 -treated patients compared with tissues following placebo treatment (135).

#### Pancreatic Cancer and Cholangiocarcinoma

The CSCs have the potential of renewal, proliferation, clonogenicity, and multipotency as adult stem cells possess (136). From the cancer stem cell theory, the dissemination of a CSC induces sustained metastatic growth which represents the primary tumor re-establishment in a secondary site (21). Recent reports suggest the CSCs partly cause the emergence of EMT, through implications from tumor stromal components. In several human cancers including pancreatic cancer, CSCs and cells with EMT-type exert a critical role in drug resistance and early metastatic process (137, 138). In Shankar’s study, RES brought the decrement of EMT markers (ZEB-1, Slug and Snail) in CSCs (21). Moreover, Qin *et al.* reported that resveratrol suppressed CSC-like properties and EMT on of pancreatic cancer cells *via* downregulating the expression of nutrient-deprivation autophagy factor-1 (NAF-1) (25).

Tumor hypoxia, a microenvironment usually found in the central part of tumors (139), promotes the process of cancer invasion and metastasis (140). Hypoxia-inducible factor-1 (HIF-1) is the most critical transcription factor of hypoxia (141). Li et al. found that RES reversed EMT by inhibiting hypoxia-mediated activation of the Hedgehog pathway and observed RES restrained the expression of HIF-1α protein as well as uPA and MMP, which are known as metastatic-related factors (22). For the above-mentioned reasons, RES may be a potential choice of prevention and treatment for pancreatic cancer.

The PI3K/AKT signaling pathway has important influence in pancreatic cancer metastasis (23). Recent studies indicate that NF-κB transcription factor might regulate the PI3K/AKT signaling pathway to trigger the metastasis-mediated effect (142). The authors show that RES reduces the expressions of p-AKT and p-NF-κB to suppress the PI3K/AKT/NF-κB pathway, inhibiting the invasion and migration of pancreatic cells as proved by suppressing the level of EMT-related markers (N-cadherin and vimentin) (23). Pancreatic stellate cells (PSCs) are usually activated and become fibroblasts in the microenvironment of desmoplastic stroma. RES is reported to block hypoxia-induced PSCs activation and inhibit the interaction between PSCs and pancreatic cancer cells (24).

Cancer associated fibroblasts (CAFs) bring about persistent inflammation and an abnormal infiltration of a desmoplastic stroma in cholangiocarcinoma (CCA) (143). CAFs also release cytokine IL-6 drive the process of EMT (144). Recently, Ferraresi *et al.* reported that IL-6 drove EMT through suppression of autophagy (145). In further researches, they found that RES could decrease the secretion (IL-6) of CAFs and make CCA cells revert N- cadherin to E-cadherin switched by induction of autophagy (LC3-II/LC3I) (26). These findings provide a new view about CAFs secretion may change the malignant phenotype of CCA cells through a functional food.

### Non-Digestive Cancers

#### Breast Cancer

TGF-β1, which is found in various tissues and has function of angiogenesis, provides cancer cells with oxygen and nutrients and becomes an immunosuppressive agent to protect tumor cells from the host immune system. So it has been linked to cell growth, invasion and metastasis in a variety of tumors (146). Tang *et al.* reported that RES could inhibit breast cancer metastasis by activating silent information regulator 7 (SIRT7) deacetylase activity toward Smad4 degradation (27), antagonizing TGF-β1 signaling. They found that mesenchymal markers involving Fibronectin 1 and vimentin, TGF-β responsive gene angiopoietin-like Protein 4 (ANGPTL4) and C-X-C motif chemokine ligand 8 (CXCL8) were significantly down-regulated by RES (27). Moreover, Sun *et al.* reported that RES could reverse TGF-β1-induced EMT of breast cancer cells by regulating PI3K/AKT and Smad signaling (28). The drugs targeting TGF-β1 ligand or receptor for the therapies of cancer are under clinical trials. However, systemic anti-TGF-β treatments might bring severe adverse effects (78). Drug development specifically targeting the TGF-β downstream signaling pathway is necessary. The findings can inspire that RES selectively targets SIRT7 and PI3K in breast cancer metastasis, which might provide new strategy for the treatment.

It is known to all that epidermal growth factor (EGF) can induce the EMT process by repressing the expression of E-cadherin, the main epithelial marker and up-regulating mesothelial markers vimentin and N-cadherin (147). González-González L. et al. reported that EGF stimulated the migratory capacity of breast cancer cells, by regulating the functional expression of NaV1.5 channels. And RES can also affect Na+ channel activity to prevent the stimulatory actions of EGF (29). Vergara D et al. also found that RES could repress EGF-induced EMT by prevention of ERK activation (30). These findings support the idea that RES prevents EGF-induced EMT through different mechanisms and can exert a preventive role in breast cancer metastasis.

Amphiregulin is high- and low-affinity ligand for EGF receptor and can maintain epithelial characteristics as efficiently as EGF (148). Cho *et al.* demonstrated that RES downregulated amphiregulin expression caused by LPA as well as blocked the Y-box binding protein-1 (YB-1)/enhancer of zeste homolog 2 (EZH2) signaling pathway to inhibit the invasion of breast cancer cell. They found EZH2 was associated with amphiregulin expression and overexpression of YB-1 increased EZH2 expression (31). Furthermore, Hartman *et al.* delineated that EZH2 was participated in NF-κB activation in triple negative breast cancer cells (TNBCs) and was critical for anchorage-independent growth of TNBCs (149). These phenomena reveal that RES has the potential to hamper EMT stimulated by LPA through suppression of YB-1/EZH2 signaling axis in breast cancer cells, preventing the invasion and metastasis, as well as sensitizing the treatment of 5-FU (31).

Interestingly, more new mechanisms are found to reverse EMT by RES. Rad9 belongs to the DNA damage response protein family, and inhibition of Rad9 can cause the corresponding downregulation of E-cadherin, and the upregulation of N-cadherin and vimentin. A research has shown that Rad9 could directly bind the Slug promoter and repress its transcriptional activity to inhibit EMT (150). Chen *et al.* found that RES could induce DNA damages and elevate ROS levels, which in turn upregulate Rad9, and suppress Slug expression to inhibit the EMT process in breast cancer cells (32). The alternative splicing of specific pre-RNAs, involving Cd44, Enah and fibroblast growth factor receptor 2 (FGFR2), exert a causative role in the EMT process (151–153). RES promotes the epithelial-type alternative splicing of Cd44, Enah, and FGFR2 pre-mRNAs in breast cancer cells by upregulating KH-type splicing regulatory protein (KHSRP) and heterogeneous nuclear ribonucleoproteins A1 (hnRNPA1) expression (33).

Doxorubicin (DOX) is one of the most commonly used chemotherapy drugs for patients of breast cancer. However, drug resistance to DOX, partly due to the occurrence of EMT, can lead to recurrence and metastasis of cancer (154). Jin et al. reported that RES successfully alleviated cell invasion and increased synergistic sensitivity to DOX *via* inhibiting the EMT process and modulating the association between silent information regulator 1 (SIRT1) and β‐catenin. They found that DOX could increase expression of vimentin, N-cadherin, β‐catenin and RES notably antagonized these EMT-markers, which showed that RES overcame DOX resistance and offered hope for breast cancer patients to extend their survival time (34). Tamoxifen, as a selective estrogen receptor (ER) modifier, is also widely used in patients with breast cancer. However, tamoxifen resistance contributes to a poor prognosis for breast cancer. Shi et al. reported that an increased production of endogenous TGF-β1 and constitutive activation of Smad signaling contributed to the cancer cell phenotype switch, meanwhile decreased the sensitivity to tamoxifen (35). The good news was that RES inhibited TGF-β1 secretion and reduced phosphorylation of Smad, eventually affecting the EMT process and reversing tamoxifen resistance (35). Targeting AKT can provide an important approach for cancer therapy and AKT inhibitor is widely studied. However, the clinically used AKT inhibitor has the side effect of promoting EMT, which might be part of drug resistance. Li et al. reported that RES could promote the β-transducin repeat containing protein (β-TrCP)-mediated Twist1 degradation and reduce MK-2206 (AKT inhibitor)-induced EMT (36). In addition, Yar et al. found that resveratrol could enhance the sensitivity of FL118 in triple-negative breast cancer cell lines by inhibiting EMT (37). It was reported that resveratrol decreased mammary promoter hypermethylation of the tumor suppressor gene Ras association domain family 1α (RASSF-1α) in women at increased risk for breast cancer (155).

#### Lung Cancer

Previous reports have pointed out that mitochondrial dysfunction has been linked to cancer metastasis (156). During the process of TGF-β1 induced EMT, mitochondria will be damaged. Under the treatment of TGF-β1, ROS level increase, while mitochondrial membrane potential and ATP content significantly decrease. Furthermore, the authors found that mitochondrial dysfunction promoted the expression level of vimentin and α-SMA, as well as decreased the expression level of E-cadherin and CK18. On the contrary, RES can protect mitochondrial function shown by an elevation in mitochondrial membrane potential, expression level of mitochondrial complex, and ATP content, so as to inhibit the occurrence of EMT (38). Wang et al. found that RES increased the expression of E-cadherin inhibited by TGF-β1 and decreased expression of fibronectin and vimentin, meanwhile RES also reduced the level of TGF-β1-induced Snail1 and Slug, thereby suppressing lung cancer invasion and metastasis (39).

Forkhead box C2 (FOXC2), a critical transcriptional factor that affects EMT, induces tumor angiogenesis and metastasis (157). Similarly, lung cancer patients with high expression of FOXC2 also experience a poor prognosis. A study has found that RES inhibited miRNA-520h, activated protein phosphatase 2 A/C (PP2A/C), then through the AKT-NF-κB signaling axis, finally down-regulated FOXC2 to inhibit distant metastasis of lung cancer cells (40).

Sun et al. found that SIRT1 transcription was regulated by a SUMOylation-dependent pathway, leading to lung cancer metastasis. Activation of SIRT1 can inhibit the occurrence of EMT. However, hypoxia repressed the transcription of SIRT1 in a SUMOylation-dependent manner. RES, the agonist of SIRT1, can antagonize the transformation of SIRT1 caused by hypoxia, thus inhibiting EMT in lung cancer cells (41).

#### Genitourinary Malignancy

A study reported that RES suppressed Snail expression and prevented cisplatin-induced EMT through the MAPK/ERK pathway in ovarian cancer cells. Meanwhile, RES can induce cell death and enhance the cytotoxicity of cisplatin. These findings suggest that RES combined with cisplatin may be a more effective antitumor regimen (42). Previous study has shown that human telomerase reverse transcriptase (hTERT) upregulated in tumor cells and is related to cancer development and poor prognosis. Noradrenaline (NE) can induce the expression of hTERT, and overexpression of hTERT can promote the expression of slug, thus speeding up the process of EMT (158). And RES happens to block the process by which NE causes EMT. Authors found that RES inhibited Src phosphorylation and HIF-1α expression, downregulating hTERT expression induced by NE. At the same time, RES also can reduce Slug expression and inhibit EMT of ovarian cancer cells. Moreover, the authors demonstrated that RES could enhance the effect of β adrenergic receptor inhibitors on NE-induced invasion of ovarian cancer cells (43). These results provide preclinical evidence for the effectiveness of RES in the treatment of ovarian cancer. Furthermore, it was reported that resveratrol could reverse EMT of cervical cancer cell *via* inhibiting STAT3^Tyr705^ phosphorylation (44).

It has been reported that changes of Mg^2+^ homeostasis affected cell functions, such as proliferation, invasion, migration, and angiogenesis (159). The transient receptor potential melastatin-subfamily member 7 (TRPM7) plays, as an Mg^2+^ influx channel, exerts tumor-promoting effects in prostate cancer. With the addition of TGF-β, the expression of TRPM7 increases, and TGF-β/TRPM7 axis is proved as one of the mechanisms of EMT. Thus, RES was found to inhibit the expression of TRPM7 induced by TGF-β, thereby inhibiting the occurrence of EMT (45). Khusbu et al. studied an unconventional E3 ligase named TNF-receptor associated factor 6 (TRAF6) and found that RES could inhibit growth and proliferation of prostate cancer cells by regulating the expression of TRAF6, suppressing the EMT process through NF-κB/Slug axis (46). Moreover, Li *et al.* found that RES could prevent LPS-induced EMT through the Hedhehog signaling pathway in prostate cancer cells (47). It is well known that prostate cancer can be easily affected by dihydrotestosterone (DHT) *via* androgen receptor (AR). Jang et al. reported that RES could prevent the metastasis of prostate cancer and reverse its process of EMT through AR and CXCR4 pathway (48). Hepatocyte growth factor (HGF), secreted by the stroma, can bind its receptor c-Met which is located in the epithelium, promoting cell growth and scattering of various epithelial cells. RES was found to suppress migration and invasion of prostate cancer epithelial cells through inhibiting HGF secretion from prostate stromal cells and to prevent the process of EMT (49).

Cigarette smoke (CS) is a main cause of bladder cancer. Sun et al. used cigarette smoke extract (CSE) to induced EMT and CSE can also promote the phosphorylation of signal transducer and activator of transcription 3 (STAT3) and the expression of Twist1, leading to the occurrence of EMT and cell invasion and metastasis. RES can promote the expression of E-cadherin and ZO-1, and reduce the expression of N-cadherin and vimentin through STAT3/Twist signaling pathway. These findings provide new ideas for clinical prevention and intervention of bladder cancer metastasis (50).

#### Brain Cancer

Song et al. reported that RES can inhibit TGF-β1-induced EMT process, migration and invasion ability in glioblastoma cells. They found that RES could downregulate EMT-related markers by inhibiting the phosphorylation of Smad2 and Smad3 which are the downstream of TGF-β1, thereby preventing the progress of EMT. And they also found that RES could inhibit the expression of cancer stem cell markers Bmil and Sox2, inhibit the stem cell-like traits of glioblastoma cells, and affect the self-renewal capacity of the cells. These findings provide experimental evidence of RES for glioblastoma treatment (51). In addition to the TGF-β1/Smad signaling axis, RES can also affect the proliferation and motility of glioma stem cells through the Wnt signaling pathway. It can also reduce the level of nuclear β-catenin and induce the transcriptional up-regulation of MYC. Finally, RES down-regulated the ribose of nuclear Twist1 and Snail1, indicating its effects as a novel anti-EMT agent (52).

A previous study demonstrated that cyclin B1 (CCNB1) can function as the control of the G2/M phase in the cell cycle (160). In addition, they found that patients with invasive pituitary adenomas share higher CCNB1 expression than these with non-invasive pituitary adenomas. Li et al. found that the knockdown of CCNB1 decreased the expression of N-cadherin, but increased the expression of E-cadherin and p120-catenin. Further analysis indicated that RES inhibited the expression level of CCNB1, regulated the proliferation and apoptosis of pituitary tumor cells, and changed the expression level of various EMT markers (53).

#### Head and Neck Cancer

More and more evidence has manifested that head and neck cancer has a subpopulation of cells which possess stem-like properties and ability to trigger tumor metastasis as well as the resistance of treatment (161). Cancer researchers pay huge attention to stemness signature interacted with EMT. For example, Hu et al. reported that RES could down-regulate the expression of Oct4, Nanog, and Nestin identified as stemness genes signatures and downregulate EMT markers (Slug, ZEB1, N-cadherin and vimentin) (54). Because of the capability to eliminate stem-like and EMT properties, RES may be a valuable therapeutic candidate. Additionally, researchers also found RES prevented the expression of Smad2/3 and down-regulated EMT markers but induced the expression of E-cadherin in oral squamous cell carcinoma (OSCC) (55). Rab coupling protein (RCP) promotes OSCC metastasis *via* elevating the expression level of ZEB1 and thereby upregulating membranetype 1 matrix metalloproteinases (MT1-MMP) expression, and this observation can be reversed by RES, revealing the potential therapeutic effects of RES on OSCC (56). Shen et al. proved that RES impeded EMT through p53 activation in CSCs of nasopharyngeal carcinoma (57).Conclusively, RES may give beneficial information for the new therapies for head and neck cancer treatment.

#### Melanoma, Osteosarcoma and Multiple Myeloma

Chen et al. found that RES exerted the antitumor effects in the metastasis melanoma mouse model, including reducing the tumor size and prolonging the survival. RES prevents tumor metastasis caused by LPS and inhibits the expression of EMT markers *via* the downregulation of NF-κB activity (58). In another study, Menicacci et al. proved that RES prevented MRC5 fibroblast senescence-associated secretory phenotype (SASP)-related protumoral effects on melanoma cells and inhibited the ability of EMT (59). In osteosarcoma cells, Sun et al. found that RES promoted the degradation of HIF-1α protein, abrogating the influence of hypoxia on proliferation, invasion and EMT (60). In conclusion, RES represents a bright prospect in the therapeutic strategies of cancers. A phase 2 study for patients with relapsed and or refractory multiple myeloma (MM) showed that the combination between SRT501 (resveratrol) and bortezomib exerted better overall response rate than SRT501 monotherapy, and renal failure seemed specific to MM patients (162).

## Resveratrol Analogs

Recently, some analogs of RES have been constructed with enhanced anti-tumor effectiveness, bioavailability, and pharmacokinetic characteristics (163). Here, we reviewed several RES analogs which were associated with EMT in different cancers. Daniele et al. developed trans-restricted analogues in which the C–C double bond of RES is displaced by diaryl substituted imidazole analogues and studied anti-tumor effects of these analogues on ovarian cancer cells. Authors found these analogues blocked the AKT and MAPK signaling, inhibiting IL-6 and EGF-induced EMT in ovarian cancer cells. These findings indicate the potential of analogues in the fight against tumor metastasis (61).

3,5,4′-Trimethoxystilbene (MR-3), a natural methoxylated analog of RES, changes the EMT-related phenotypes more effectively than RES in MCF-7 cells (62). Authors found that MR-3 could restore membrane-bound β-catenin as well as suppress the nuclear localization and function of β-catenin in breast cancer cells. Further, MR-3 significantly decreases the phosphorylation of GSK-3β and the phosphorylated status of AKT is blocked. Altogether, MR-3 reverses the occurrence of EMT through the degradation of PI3K/AKT/GSK-3β signaling and the synthesis of β-catenin proteasome. Simultaneously, this finding suggests that methoxylation may increase the EMT-inhibiting effect of RES.

Another RES analog, Triacetyl-resveratrol (TCRV), which provides a better pharmacokinetics/pharmacodynamics traits than parent RES, was studied in pancreatic cancer cells by Junsheng Fu and his team (63). Authors found that TCRV inhibited EMT by suppressing the expression of ZEB1 *via* the upregulation of miR-200 family members and by the suppression of the sonic hedgehog (Shh) pathway. These findings suggest TCRV might be a promising candidate for the prevention and treatment of pancreatic cancer.

## Conclusions and Perspectives

More and more studies have demonstrated that the EMT process exerts a significant role on tumor progression and metastasis (7–9). The EMT program is associated with various pathways and crosstalk between these signaling by key molecules. Interestingly, EMT induction promotes the CSC characteristics and contributes to recurrence and drug resistance. TGF-β1 has widely been regarded as an effective inducer of the EMT process *via* both Smad-dependent and other signaling. RTK pathways also play essential roles in the process of EMT. Thus, therapies targeting these signaling pathways might provide promising approaches to inhibit invasion of cancer cells and to prevent metastasis. RES has attracted increasing attention in recent 20 years with its impact on preventing cancer and its low toxicity for normal cells *in vivo*. In addition, the safety of oral administration has also been proved by many preclinical and clinical studies. Because of its low water-solubility and bioavailability, some RES analogs, such as 3,5,4’-Trimethoxystilbene and Triacetyl resveratrol, are constructed. Several studies have confirmed that RES and its analogues inhibit cancer cell growth, invasion, migration as well as promote the apoptosis of tumor cells. RES can also reverse and prevent EMT, increasing cell-cell tight junctions and eventually inhibiting the metastasis of cancers. Recent data reveal that RES can show anti-tumor effects by suppression of EMT *via* both TGF-β1-dependent (e.g. breast cancer, lung cancer and glioblastoma multiforme) and -independent (e.g. gastric cancer, pancreatic cancer, ovarian cancer, melanoma and osteosarcoma) signaling pathways. Moreover, it has been reported that RES and its analogs can abolish chemoresistance through EMT inhibition and improve the antiproliferative effects of conventional treatments (e.g. doxorubicin, 5-FU, tamoxifen and cisplatin). Therefore, RES and analogs have the potential ability to be applied as novel complementary and alternative agents to hamper cancer invasion and metastasis, and it may partly be attributed to their effectiveness to prevent EMT.

## Author Contributions

Content design, KG, YF, XZ, LS, HW, MS and SR. Literature collection, KG, YF and XZ. Arrangement, KG. Figures and tables, KG and YF. Writing-original draft, KG, YF and XZ. Writing—review and editing, KG, YF, XZ, LS, HW, MS and SR. Writing—proofreading, LS. Funding, SR. All authors contributed to the article and approved the submitted version.

## Funding

National Natural Science Foundation of China (81573902); Zhejiang provincial TCM scientific research fund project (2019ZQ015); China Postdoctoral Science Foundation (2017M612040; 2018T110610); Top ten thousand talents program of Zhejiang Province (SR, no.2019-97, http://www.zjzzgz.gov.cn/); Zhejiang Provincial Project for the key discipline of Traditional Chinese Medicine (YG, no,2017-XK-A09, http://www.zjwjw.gov.cn/); Science and technology innovation activity plan and new seedling of college students in Zhejiang Province (2019R410001).

## Conflict of Interest

The authors declare that the research was conducted in the absence of any commercial or financial relationships that could be construed as a potential conflict of interest.
